# AlCl_3_ Aerosol Exposure Is Associated With Pulmonary Lactate Accumulation, Neutrophil Dysfunction, and Fibrosis‐Like Remodeling

**DOI:** 10.1002/biof.70146

**Published:** 2026-08-23

**Authors:** Jiayu Qin, Fushi Dong, Zhengjie Ye, Xiangxin Yao, Xintong Wang, Tiquan Xiao, Jing Yang, Yan Zheng, Yonggang Cao, Chunli Che

**Affiliations:** ^1^ Respiratory and Critical Care Medicine Department The Fourth Affiliated Hospital of Harbin Medical University Harbin China; ^2^ Department of Respiratory Medicine The First Affiliated Hospital of Harbin Medical University Harbin China; ^3^ College of Basic Medicine, Harbin Medical University Harbin China; ^4^ The Fifth Affiliated Hospital of Harbin Medical University Daqing China

**Keywords:** AlCl_3_ aerosol exposure, fibrosis‐like remodeling, lactate, MCT1, neutrophil dysfunction, translational medicine

## Abstract

Aluminum‐associated interstitial lung disease is an exposure‐related fibrotic lung disorder, but the metabolic and innate immune mechanisms underlying persistent pulmonary remodeling after aluminum exposure remain incompletely understood. We investigated whether lactate‐associated neutrophil dysfunction contributes to lung injury and fibrosis‐like remodeling following AlCl_3_ aerosol exposure. Rats were subjected to repeated AlCl_3_ aerosol exposure with or without pharmacological inhibition of lactate transport using the monocarboxylate transporter 1 inhibitor AZD3965. Pulmonary injury, lactate accumulation, inflammatory responses, oxidative stress, DNA damage response signaling, and fibrosis‐like remodeling were assessed. Bone marrow‐derived rat neutrophils were treated with sodium L‐lactate in the presence or absence of AZD3965 to evaluate chemotaxis, oxidative burst, mitochondrial membrane potential, calcium signaling, intracellular lactate accumulation, and neutrophil extracellular trap (NET)‐associated responses. In addition, a supportive human cohort comprising patients with aluminum exposure‐related interstitial lung disease (Al‐ILD), patients with nonaluminum interstitial lung disease (non‐Al‐ILD), and healthy controls was analyzed using quantitative chest computed tomography (CT), pulmonary function testing, and peripheral blood transcriptomics. Repeated AlCl_3_ aerosol exposure was associated with pulmonary aluminum deposition, lactate accumulation, inflammatory injury, oxidative stress, DNA damage response signaling, neutrophil abnormalities, and collagen‐rich fibrosis‐like remodeling in rats. In vitro, high‐lactate‐associated metabolic stress impaired neutrophil chemotaxis, oxidative burst, mitochondrial membrane potential, calcium signaling, and NET‐associated responses. AZD3965 reduced intracellular and pulmonary lactate accumulation and partially ameliorated neutrophil dysfunction, inflammatory injury, oxidative stress, and fibrosis‐like remodeling in experimental models. In the supportive human cohort, patients with Al‐ILD showed greater quantitative CT‐defined parenchymal abnormality burden and lower forced vital capacity and diffusing capacity for carbon monoxide than patients with non‐Al‐ILD. Peripheral blood transcriptomic analysis revealed enrichment of innate immune, cytokine signaling, extracellular matrix organization, and NET‐related pathways in Al‐ILD. Collectively, these findings indicate that AlCl_3_ aerosol exposure is associated with pulmonary lactate accumulation, neutrophil dysfunction, and fibrosis‐like remodeling. Pharmacological modulation of lactate transport provides supportive evidence for the involvement of lactate‐associated metabolic regulation in this process, although further studies are required to define the specific molecular targets involved. The integration of experimental and supportive human findings suggests that the lactate–neutrophil dysfunction axis may represent a mechanistically relevant feature of aluminum‐associated lung injury and remodeling.

## Graphical abstract

AlCl_3_ aerosol exposure drives pulmonary lactate accumulation, impairing neutrophil ROS production, chemotaxis, and NET‐associated responses and promoting fibrosis‐like lung remodeling. Pharmacological blockade of MCT1‐mediated lactate transport with AZD3965 may offer a potential intervention strategy.

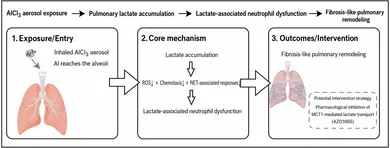

**Abbreviations**
Al: aluminum
Al‐ILD: aluminum exposure‐related interstitial lung disease
DLCO: diffusing capacity for carbon monoxide
FVC: forced vital capacity
ILD: interstitial lung disease
MCT1: monocarboxylate transporter 1
NET: neutrophil extracellular trap
ROS: reactive oxygen species

## 1 Background

Metal constituents of fine particulate matter (PM2.5) are increasingly recognized as contributors to exposure‐related lung injury and chronic respiratory morbidity. Aluminum (Al) is among the predominant metallic contaminants in PM2.5 [1] and has been associated with occupational and nonoccupational pulmonary disorders, including interstitial lung abnormalities and progressive fibrotic remodeling [2, 3]. Although aluminum‐induced lung injury has been associated with oxidative stress and pulmonary toxicity [4, 5], the mechanisms linking aluminum exposure to persistent tissue remodeling remain incompletely defined. In particular, whether Al exposure reshapes the pulmonary metabolic microenvironment and disrupts innate immune effector programs during fibrotic progression is unclear.

Pulmonary fibrosis is characterized by excessive extracellular matrix deposition and irreversible distortion of lung architecture [6, 7]. In addition to inflammatory and oxidative injury, increasing evidence indicates that metabolic and redox remodeling contribute to fibrotic progression [8]. Oxidative stress is a central amplifier of fibroblast activation, whereas antioxidant systems such as the glutathione axis critically modulate fibrotic susceptibility [9, 10]. These observations suggest that exposure‐induced metabolic–redox dysregulation may represent a pathogenic route linking environmental injury to aberrant repair and fibrosis.

Neutrophils are increasingly appreciated as active regulators of fibrotic lung disease rather than passive bystanders. Their effector functions, including oxidative burst, migration, and neutrophil extracellular trap (NET) formation, are tightly coupled to intracellular metabolic and redox status [11, 12]. Persistent dysregulation of neutrophil responses may therefore amplify tissue injury and alter repair trajectories, and NET‐associated signatures have been linked to fibrotic lung disease severity [13].

Lactate, once regarded as a terminal glycolytic by‐product, is now recognized as a central immunometabolic signal that modulates immune‐cell activation, migration, and effector functions [14]. Lactate is transported across cell membranes by proton‐linked monocarboxylate transporters, including MCT1/SLC16A1 [15, 16]. Extracellular lactate availability can thereby influence intracellular metabolic and immune‐cell programs [16]. In fibrotic lung disease, lactate is elevated in diseased tissues and can promote profibrotic myofibroblast differentiation through pH‐dependent activation of latent transforming growth factor‐β [17]. These findings position lactate as a plausible, but not definitive, metabolic intermediate linking exposure‐associated metabolic stress to immune dysfunction and fibrotic remodeling.

Nevertheless, whether aluminum exposure is accompanied by a lactate‐enriched pulmonary microenvironment that alters neutrophil function remains unknown. Mechanistically, evaluating the relationship between lactate flux and neutrophil dysfunction requires interrogation of lactate transport pathways, for which the MCT1 inhibitor AZD3965 provides a tractable pharmacological tool [15]. In parallel, exposure‐related tissue injury may be reflected by genomic stress responses, and γ‐H2AX is a sensitive marker of DNA double‐strand break‐associated response signaling [18].

We hypothesized that AlCl_3_ aerosol exposure is associated with pulmonary lactate accumulation and altered neutrophil immunometabolic homeostasis, which may contribute to fibrosis‐like remodeling. To test this hypothesis, we integrated an experimental AlCl_3_ aerosol exposure model, in vitro neutrophil functional assays, and supportive human data to evaluate the relevance of a lactate‐associated neutrophil dysfunction axis in AlCl_3_ aerosol exposure‐related lung injury.

## 2 Materials and Methods

### 2.1 Animal Exposure Model and Intervention

Eight‐week‐old male Wistar rats (160–200 g) were housed under standardized specific‐pathogen‐free conditions with free access to food and water. An aluminum trichloride (AlCl_3_) aerosol exposure model of lung injury was established using a whole‐body exposure system. AlCl_3_ aerosol was generated using an ultrasonic nebulizer and delivered into the exposure chamber. The reported aerosol concentrations were expressed as elemental aluminum equivalents (mg Al‐equivalent/m^3^), rather than as the total mass concentration of AlCl_3_. Aerodynamic particle‐size distribution was not measured contemporaneously during the original animal exposure sessions; therefore, the experimental aerosol was not classified as PM2.5. Because contemporaneous aerosol concentration measurements at the animals' breathing zone could not be verified from the available study documentation, the reported values are described as target concentrations. A 7‐day dose‐screening experiment using target concentrations of 0.5, 1.0, 2.0, and 4.0 mg Al‐equivalent/m^3^ for 4 h/day identified 2.0 mg Al‐equivalent/m^3^ as a robust but tolerable target concentration, whereas 4.0 mg Al‐equivalent/m^3^ caused more extensive lung injury. Therefore, a target concentration of 2.0 mg Al‐equivalent/m^3^ was selected for the subsequent 4‐week experiment. The target concentration was selected with reference to previous inhalational aluminum studies and was intended to model repeated AlCl_3_ aerosol exposure under controlled experimental conditions [19]. Rats were randomly assigned to the Sham, AlCl_3_, or AlCl_3_ + AZD3965 groups (*n* = 12 per group). Animals in the Sham group were exposed to aerosolized sterile normal saline (0.9% NaCl) generated using the same ultrasonic nebulizer and delivered under otherwise identical chamber conditions, exposure duration, and exposure schedule as those used for the AlCl_3_‐treated groups. Animals in the exposure groups underwent AlCl_3_ aerosol exposure for 4 h/day, 5 days/week, for 4 consecutive weeks. AZD3965 was administered intraperitoneally at 100 mg/kg 30 min before each AlCl_3_ aerosol exposure. The dose, route, and pretreatment interval were selected with reference to previously reported AZD3965 pharmacological studies and adjusted for this proof‐of‐concept experiment [20]. Because this regimen was established in a tumor‐bearing mouse model rather than a rat inhalational lung‐injury model, it was used as a pharmacological proof‐of‐concept and interpreted cautiously. Because different assays required distinct and, in some cases, incompatible tissue‐processing procedures, not all animals were used for every endpoint. Animals and tissue samples were allocated to individual analyses according to a predefined scheme established before outcome assessment.

Unless otherwise specified, quantitative biochemical analyses included six independent animals per group, whereas Western blot analyses included three independent animals per group. The reported n values represent independent animals. No animal, sample, or data point was excluded on the basis of the observed results, and the actual sample size for each analysis is provided in the corresponding figure legend.

Sample sizes were based on the dose‐screening experiment, previous inhalational aluminum‐exposure studies, and feasibility considerations. All animal procedures were approved by the Institutional Animal Care and Use Committee of Harbin Medical University (approval no. HMUDQ 20251221003) and were conducted in accordance with institutional guidelines for the care and use of laboratory animals.

### 2.2 Histological and Immunohistochemical Analyses

Frozen lung sections were subjected to lumogallion staining (GC49604, GLPBIO, USA) for aluminum deposition, hematoxylin and eosin (H&E) staining for histopathology, Masson's trichrome staining (G1340; Solarbio, China) for collagen deposition, and TUNEL staining (G4891, Solarbio, China) for apoptosis, following manufacturer protocols. Immunofluorescence staining was performed for γ‐H2AX, MPO, and CitH3 (AP0687, A17562, Abclonal; 22225–1‐AP, Proteintech, China), with DAPI (C1006, Beyotime, China) nuclear counterstaining.

### 2.3 Inflammatory and Injury Assessments

Peripheral blood neutrophil percentages were determined using an automated hematology analyzer (Sysmex XN‐1000). Lung wet‐to‐dry (W/D) ratios were calculated to assess pulmonary edema. Lung tissue levels of IL‐1β, IL‐6, and TNF‐α (EK0393, EK0412, EK0526; Boster, China) were measured by ELISA according to manufacturer instructions.

### 2.4 Metabolomic and Biochemical Assays

Untargeted metabolomic profiling of lung tissue was performed using UHPLC–QTOF‐MS in both positive and negative ion modes. Data were processed for peak alignment, metabolite identification, and relative quantification.

Lactate concentrations in lung tissue and serum, as well as intracellular lactate levels in neutrophils, were quantified using an L‐lactate assay kit (BC2230, Solarbio, China). Antioxidant status was assessed by measuring GSH content (S0053, Beyotime, China), glutathione peroxidase (GSH‐Px) activity (S0056, Beyotime, China), superoxide dismutase (SOD) activity (S0101S, Beyotime, China), and malondialdehyde (MDA) levels using commercial kits (S0131S, Beyotime, China).

### 2.5 Neutrophil Isolation and in Vitro Treatments

Rat bone marrow neutrophils were isolated using a density‐gradient‐based commercial kit (P9200, Solarbio, China). For the in vitro functional experiments, neutrophils were assigned to the Control, Lactate, and Lactate + AZD3965 groups. Cells in the Lactate and Lactate + AZD3965 groups were treated with 20 mM sodium L‐lactate, and cells in the Lactate + AZD3965 group additionally received 1 μM AZD3965. The pH of all treatment media was adjusted to 7.4. Cell viability was assessed using an MTT assay (M1020; Solarbio, China). Because lactate at 20 mM may represent a high‐lactate or supraphysiological stress condition, functional readouts were interpreted together with MTT results to reduce overinterpretation of nonspecific cytotoxic or metabolic effects. Although pH was controlled, potential confounding effects related to osmotic pressure, sodium load, metabolic stress, and altered cellular energy status were considered in the interpretation of the in vitro findings.

### 2.6 Functional Assays of Neutrophils

Neutrophil chemotaxis was evaluated using Transwell chambers (5 μm pore size). NET formation was assessed by transmission electron microscopy (TEM) and immunofluorescence staining for MPO and CitH3. MPO and CitH3 protein expression was assessed by Western blot analysis.

Mitochondrial membrane potential was measured using JC‐1 staining (M8650; Solarbio, China). Intracellular reactive oxygen species (ROS) were detected using DCFH‐DA fluorescence (F8500; Solarbio, China). Intracellular Ca^2+^ signaling was assessed with Fluo‐4 AM (IF1500; Solarbio, China).

### 2.7 Pulmonary Microcirculation Imaging

Laser speckle contrast imaging (Moor FLPI‐2, UK) was used to assess pulmonary surface blood flow following thoracotomy and mechanical ventilation, as previously described by Shi et al. [21] and Yang et al. [22].

### 2.8 Supportive Human Relevance Cohort

A single‐center prospective cohort was established at the Department of Respiratory Medicine, the Fourth Affiliated Hospital of Harbin Medical University, between March 2024 and March 2025, to provide supportive human relevance for the experimental findings. Interstitial lung disease (ILD) was diagnosed by multidisciplinary discussion according to the 2022 ATS/ERS/JRS/ALAT guideline criteria [23]. Aluminum exposure‐related ILD (Al‐ILD) was defined by a documented history of occupational or environmental aluminum exposure for at least 10 years in the absence of a more plausible alternative etiology. Patients with nonaluminum ILD served as disease controls, and healthy controls were recruited from the health examination center. Healthy controls were required to have normal pulmonary function, no interstitial abnormalities on chest imaging, and no respiratory infection within 4 weeks before enrollment. Exclusion criteria included age < 18 years, pregnancy or lactation, malignancy, severe heart failure with pulmonary edema, and active infection. Key baseline variables considered for interpretation included age, sex, smoking status, exposure type and duration, working ILD diagnosis, HRCT pattern, pulmonary function, and treatment status. Written informed consent was obtained from all participants prior to enrollment. Ultimately, five patients with Al‐ILD, eight patients with non‐Al‐ILD, and six healthy controls were enrolled. Quantitative chest CT and pulmonary function comparisons were performed between the Al‐ILD and non‐Al‐ILD groups. For healthy controls, available chest imaging and pulmonary function results were reviewed to confirm eligibility but were not included in the comparative analyses presented in Figure 8. Peripheral blood transcriptomic analysis was performed across all three groups. The study protocol was approved by the Ethics Committee of the Fourth Affiliated Hospital of Harbin Medical University (approval no. MR‐23‐25‐002076) and was conducted in accordance with the Declaration of Helsinki and its later amendments.

### 2.9 Pulmonary Function Testing and Quantitative CT Analysis

Pulmonary function testing was performed using a MasterScreen pulmonary function system (Jaeger, Germany). FVC was measured according to the 2019 ATS/ERS spirometry standards [24], whereas DLCO was measured according to the 2017 ERS/ATS standards for single‐breath carbon monoxide uptake [25]. Forced vital capacity (FVC) and diffusing capacity for carbon monoxide (DLCO) were recorded and expressed as percentages of predicted values. Chest CT images were quantitatively analyzed using 3D Slicer (version 5.2.2) with the Lung CT Analyzer extension for automated lung segmentation and parenchymal abnormality quantification [26, 27]. Automated segmentation results were visually reviewed by two senior radiologists blinded to clinical grouping and manually corrected when necessary. Lung regions were classified according to predefined attenuation thresholds into emphysema, normally aerated lung, infiltration, and collapsed lung compartments. The infiltration and collapsed lung compartments were combined as an operational measure of increased parenchymal abnormality burden rather than a direct histopathological classification. Quantitative CT measurements and pulmonary function parameters were formally compared between the Al‐ILD and non‐Al‐ILD groups to evaluate differences in structural abnormality burden and functional impairment. Healthy controls had chest imaging and pulmonary function data available from routine health examinations. These data were reviewed to confirm normal pulmonary function and the absence of interstitial abnormalities, as required by the eligibility criteria. However, healthy‐control CT images were not subjected to the same quantitative CT analysis, and their imaging and pulmonary function data were not included in the statistical comparisons presented in Figure 8, because this analysis was specifically designed to compare disease severity between the two ILD groups.

### 2.10 Transcriptomic Analysis in the Supportive Human Relevance Cohort

Peripheral blood RNA sequencing was performed by Sangon Biotech (Shanghai, China) on an Illumina HiSeq platform using 150‐bp paired‐end reads. Raw reads were assessed with FastQC v0.11.2 and filtered using Trimmomatic v0.36 by removing adapters, undetermined bases, bases with quality scores < 20, and reads shorter than 35 nucleotides.

Clean reads were aligned to the Ensembl human reference genome using HISAT2 v2.1.0; the exact genome assembly and annotation release were not retained in the archived report. Gene‐level read counts and TPM values were estimated using StringTie v1.3.3b. Differential expression was analyzed using DESeq2 v1.12.4 for the three pairwise comparisons, with significant DEGs defined as |log_2_ fold change| > 1 and FDR‐adjusted *q* < 0.05.

Figure 9D–F was based on DEGs from Al‐ILD versus non‐Al‐ILD. PPI analysis used STRING and igraph v1.0.1, whereas GO and KEGG enrichment analyses used topGO v2.24.0 and clusterProfiler v3.0.5, respectively, with *q* < 0.05 considered significant. For Figure 9G, DEG lists from Al‐ILD versus healthy controls and non‐Al‐ILD versus healthy controls were separately analyzed in Metascape on December 20, 2024, for *Homo sapiens* using default settings.

### 2.11 Statistical Analysis

Statistical analyses were performed using OriginPro 2025. Data are presented as mean ± SEM. Normality and homogeneity of variance were assessed using the Shapiro–Wilk and Levene's tests, respectively. Two‐group comparisons were performed using a two‐tailed unpaired Student's *t* test, and multiple‐group comparisons were conducted using one‐way ANOVA followed by Tukey's post hoc test. Transcriptomic analyses were adjusted for multiple testing using false discovery rate correction. A *p* < 0.05 was considered statistically significant.

For animal studies, *n* represents independent animals; each Western blot biological replicate was derived from a different animal. For in vitro studies, *n* represents independent biological experiments, with technical replicates averaged to generate one value per experiment. For clinical studies, n represents independent participants. Animals and tissues were allocated to assays before outcome assessment. No outcome‐based exclusions or post hoc outlier removal were performed, and the actual sample size is reported in each figure legend.

## 3 Results

### 3.1 AlCl_3_ Aerosol Exposure Is Associated With Pulmonary Aluminum Deposition and Fibrosis‐Like Remodeling

**FIGURE 1:**
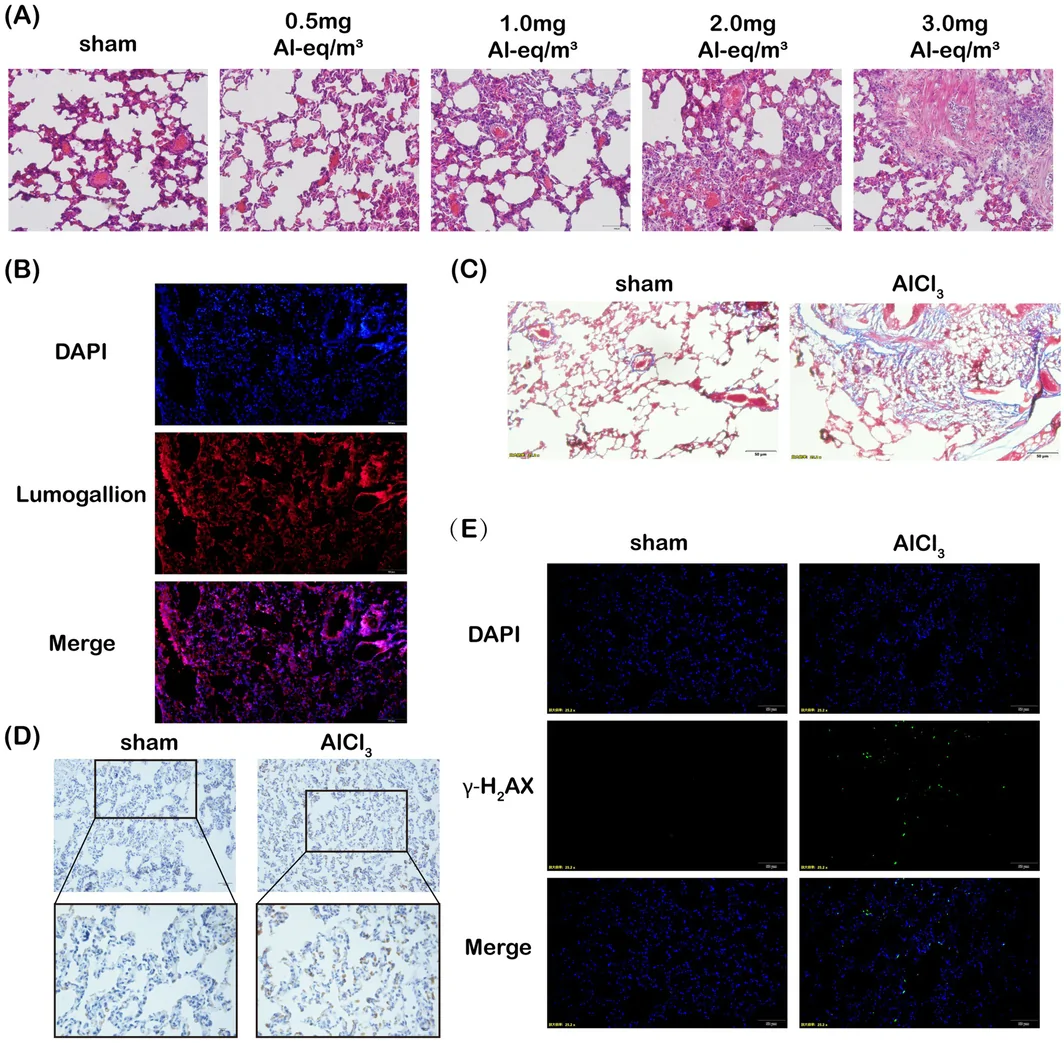
Nebulized AlCl_3_ inhalation is associated with pulmonary aluminum deposition and fibrosis‐like lung injury accompanied by cell death and DNA damage response signaling. (A) Representative H&E staining of lung sections from rats exposed to sham aerosol or AlCl_3_ aerosol at target concentrations of 0.5, 1.0, 2.0, and 4.0 mg Al‐equivalent/m^3^ in the 7‐day dose‐finding experiment (*n* = 3 animals/group). Aluminum exposure was associated with dose‐dependent lung injury, including alveolar structural disruption, septal thickening, and inflammatory cell infiltration. (B) Representative lumogallion staining of lung sections from the sham and model groups in the 4‐week exposure experiment (*n* = 3 animals/group), showing pulmonary aluminum deposition. DAPI was used for nuclear counterstaining. (C) Representative Masson's trichrome staining of lung sections from the sham and model groups in the 4‐week exposure experiment (*n* = 3 animals/group), showing increased collagen deposition after chronic AlCl_3_ inhalation. (D) Representative TUNEL staining of lung sections from the sham and model groups in the 4‐week exposure experiment (*n* = 3 animals/group), showing increased cell death in the model group. Insets indicate higher‐magnification views of the boxed regions. (E) Representative immunofluorescence staining of γ‐H2AX in lung sections from the sham and model groups in the 4‐week exposure experiment (*n* = 3 animals/group), showing increased DNA double‐strand break‐associated response signals after AlCl_3_ exposure. DAPI was used for nuclear counterstaining. Scale bars as indicated.

Rats subjected to AlCl_3_ aerosol exposure developed dose‐dependent lung injury, characterized by disruption of alveolar architecture, thickening of alveolar septa, and inflammatory cell infiltration (Figure 1A). In the 7‐day dose‐finding experiment, a target concentration of 2.0 mg Al‐equivalent/m^3^ produced robust but tolerable pathological alterations, whereas 4.0 mg Al‐equivalent/m^3^ was associated with more extensive tissue injury; therefore, 2.0 mg Al‐equivalent/m^3^ was selected as the target concentration for the subsequent 4‐week exposure study. In the chronic AlCl_3_ aerosol exposure model, lumogallion staining showed marked pulmonary aluminum deposition (Figure 1B), Masson's trichrome staining demonstrated increased collagen deposition (Figure 1C), TUNEL staining revealed increased cell death (Figure 1D), and γ‐H2AX immunofluorescence showed enhanced DNA double‐strand break‐associated response signals in exposed lungs (Figure 1E). Together, these findings show that AlCl_3_ aerosol exposure is associated with pulmonary aluminum accumulation accompanied by fibrosis‐like remodeling, cell death, and DNA damage response signaling.

### 3.2 AlCl_3_ Aerosol Exposure Is Associated With Neutrophilia and Pulmonary Inflammatory Responses

**FIGURE 2:**
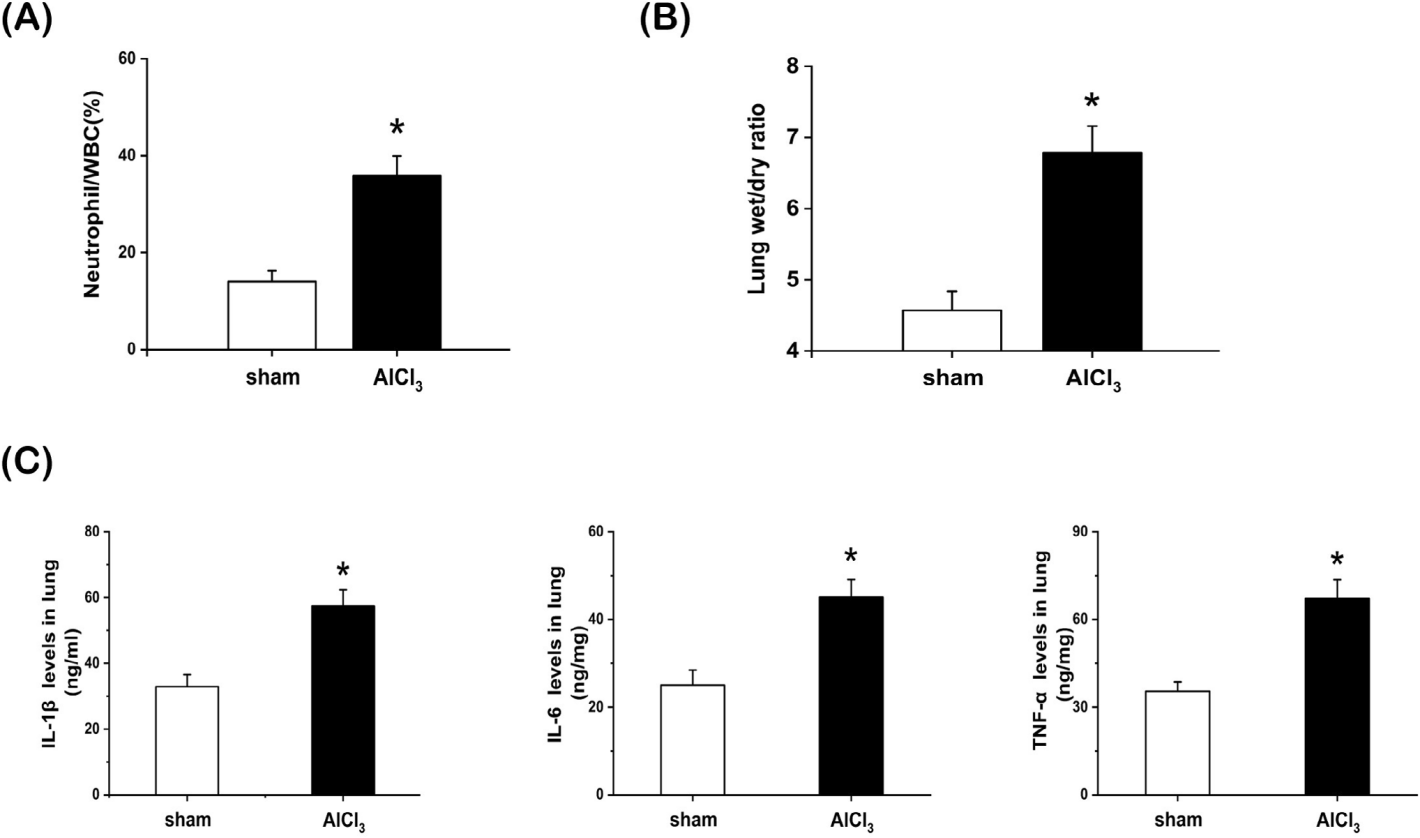
Nebulized AlCl_3_ inhalation is associated with systemic neutrophilia, pulmonary edema, and proinflammatory cytokine production. (A) Percentage of neutrophils among total white blood cells in peripheral blood from the sham and model groups. (B) Lung wet‐to‐dry ratio in the sham and model groups. (C) Levels of IL‐1β, IL‐6, and TNF‐α in lung tissues measured by ELISA. For all panels, *n* = 6 animals/group. Data are presented as mean ± SEM. Two‐group comparisons were performed using a two‐tailed Student's *t* test. *p < 0.05 versus sham.

To assess inflammatory responses following AlCl_3_ aerosol exposure, peripheral blood inflammatory cells, lung wet‐to‐dry (W/D) ratios, and pulmonary cytokine levels were measured (Figure 2). Compared with the sham group, AlCl_3_‐exposed rats showed a significantly increased proportion of neutrophils among circulating white blood cells (Figure 2A) and a higher lung W/D ratio (Figure 2B). In addition, lung tissue levels of IL‐1β, IL‐6, and TNF‐α were significantly elevated after AlCl_3_ aerosol exposure (Figure 2C). These findings indicate that AlCl_3_ aerosol exposure is associated with systemic neutrophilia, pulmonary edema, and a marked proinflammatory response.

### 3.3 AlCl_3_ Aerosol Exposure Increases Pulmonary Lactate Accumulation and Reshapes Lung Metabolism

**FIGURE 3:**
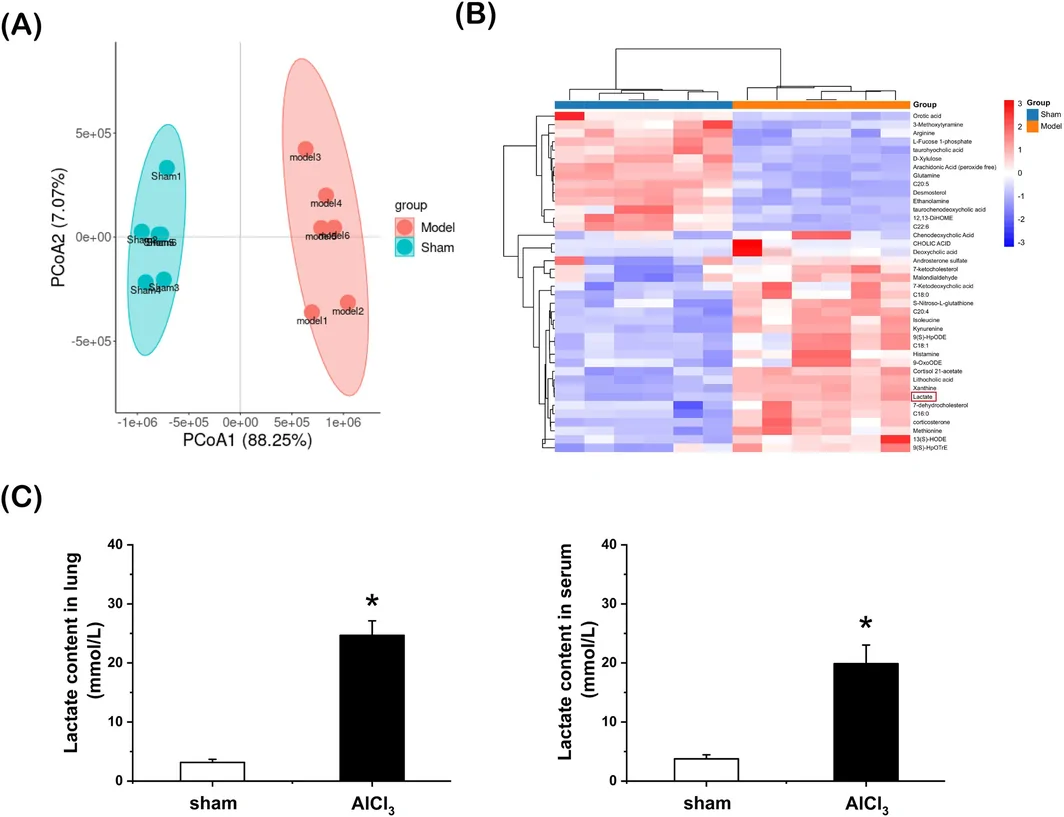
Aluminum exposure reshapes pulmonary metabolism and increases lactate accumulation in lung tissue and serum. (A) Principal coordinate analysis (PCoA) score plot showing separation of lung metabolomic profiles between the sham and model groups. (B) Hierarchical clustering heatmap showing differential metabolite patterns in lung tissues from the sham and model groups. (C) Lactate concentrations in lung tissue and serum from the sham and model groups. For all panels, *n* = 6 animals/group. Data are presented as mean ± SEM. Two‐group comparisons were performed using a two‐tailed Student's *t* test. *p < 0.05 versus sham. For untargeted metabolomics in (A and B), each point represents an individual biological sample.

Untargeted metabolomic profiling revealed marked metabolic remodeling in the lungs of AlCl_3_‐exposed rats (Figure 3). Principal coordinate analysis showed clear separation between the sham and exposed groups, indicating distinct pulmonary metabolic profiles (Figure 3A), and hierarchical clustering further illustrated differential metabolite patterns between the two groups (Figure 3B). Among the altered metabolites, lactate emerged as a prominent increased metabolite. Targeted assays further showed significantly elevated lactate levels in both lung tissue and serum following AlCl_3_ aerosol exposure (Figure 3C). Together, these findings identify lactate accumulation as a prominent metabolic feature of experimental lung injury induced by AlCl_3_ aerosol exposure.

### 3.4 Lactate‐Associated Metabolic Stress Is Accompanied by Impaired Neutrophil Oxidative Burst and Mitochondrial Dysfunction in Vitro

**FIGURE 4:**
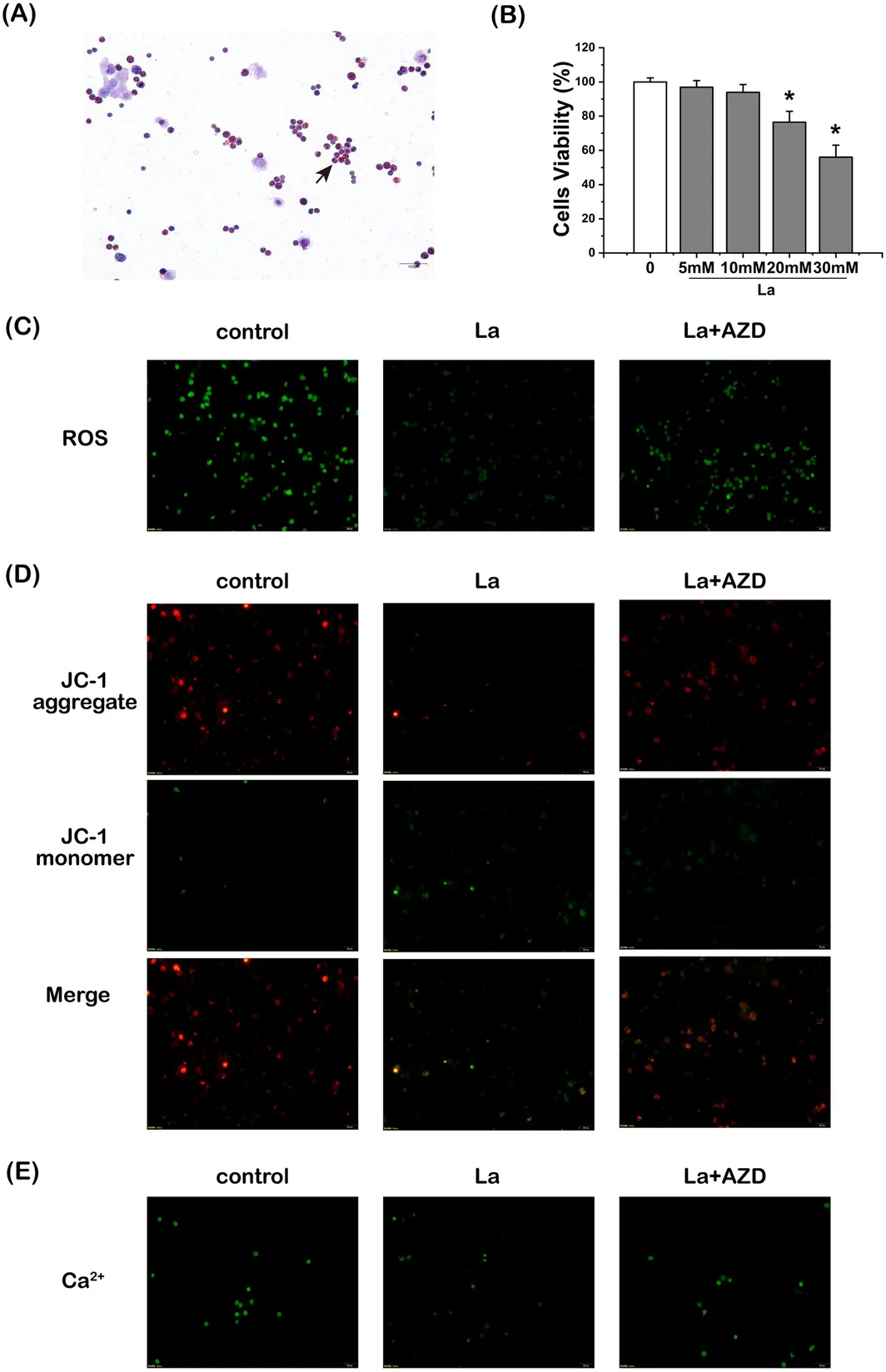
Lactate‐associated metabolic stress is accompanied by impaired neutrophil oxidative burst and mitochondrial function in vitro, which is partially ameliorated by AZD3965. (A) Representative H&E staining of isolated rat bone marrow‐derived neutrophils. (B) Neutrophil viability after treatment with increasing concentrations of lactate (La). (C) Representative fluorescence images showing ROS levels in control, La, and La + AZD3965 groups. (D) Representative JC‐1 staining showing changes in mitochondrial membrane potential in control, La, and La + AZD3965 groups. Red fluorescence indicates JC‐1 aggregates and green fluorescence indicates JC‐1 monomers. (E) Representative fluorescence images showing intracellular Ca^2+^ signals in control, La, and La + AZD3965 groups. For quantitative analyses, *n* = 3 independent biological replicates per group. Data are presented as mean ± SEM. Panel (B) was analyzed by one‐way ANOVA followed by Tukey's post hoc test. For comparisons among control, La, and La + AZD3965 groups in (C–E), one‐way ANOVA followed by Tukey's post hoc test was used for quantified data. *p < 0.05 versus control; ^#^ *p* < 0.05 versus La. Scale bars as indicated.

Rat bone marrow‐derived neutrophils were isolated and exposed to sodium L‐lactate in vitro to assess functional responses (Figure 4A). MTT assays showed a dose‐dependent reduction in neutrophil viability/metabolic activity, with significant inhibition at concentrations of 20 mM or higher (Figure 4B), supporting the interpretation of 20 mM lactate as a high‐lactate stress condition. Lactate treatment markedly reduced intracellular ROS fluorescence, whereas AZD3965 partially ameliorated oxidative burst signals (Figure 4C). Consistently, JC‐1 staining showed a reduction in mitochondrial membrane potential after lactate treatment, which was partially ameliorated by AZD3965 (Figure 4D). Intracellular Ca^2+^ fluorescence was also reduced by lactate and partially ameliorated by AZD3965 (Figure 4E). These results suggest that lactate‐associated metabolic stress is accompanied by impaired oxidative burst, mitochondrial function, and calcium homeostasis in neutrophils, with partial rescue by AZD3965.

### 3.5 Lactate‐Associated Metabolic Stress Is Accompanied by Impaired Neutrophil Chemotaxis and NET‐Associated Responses in Vitro, Which Are Partially Ameliorated by AZD3965

**FIGURE 5:**
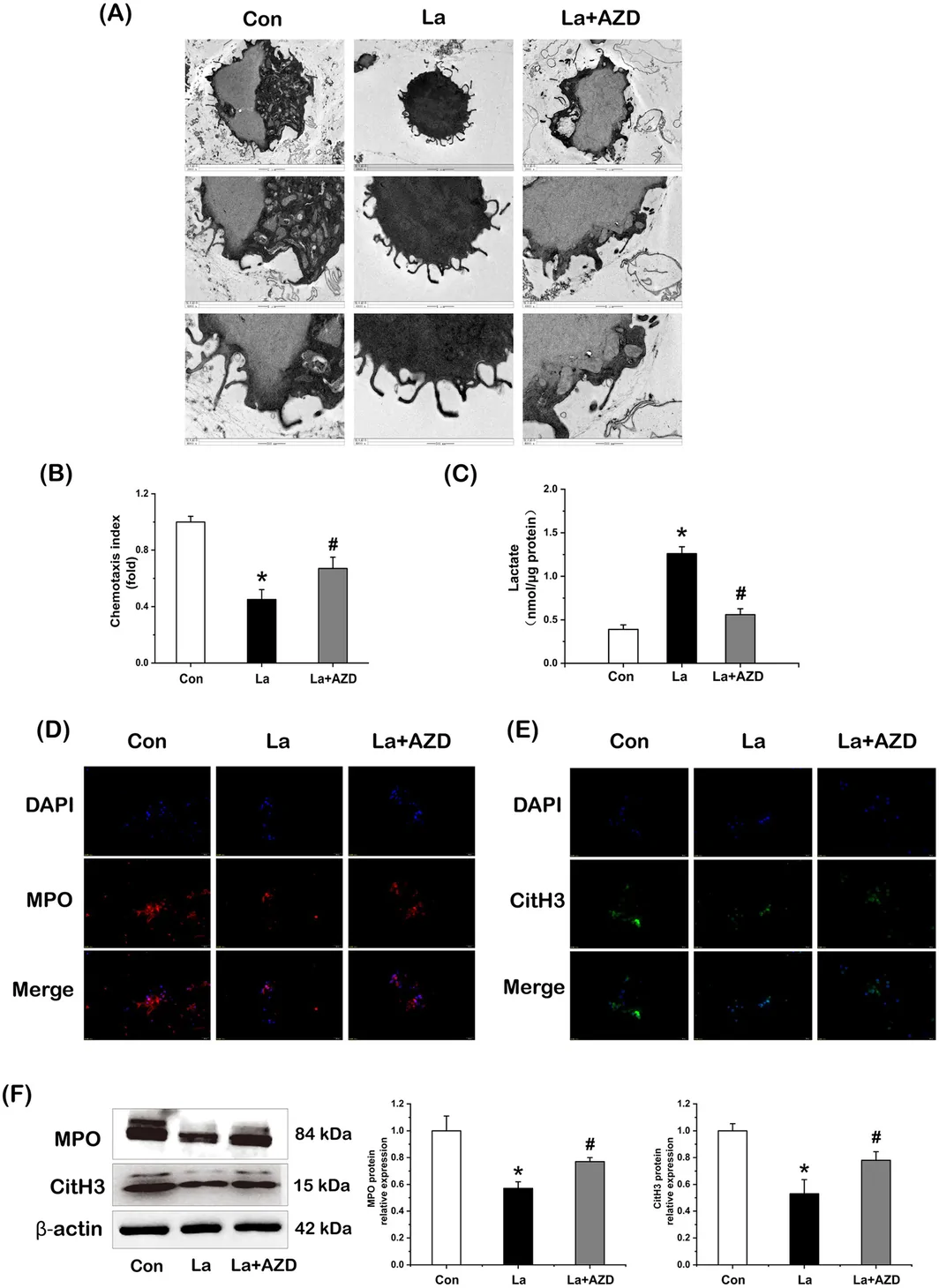
Lactate‐associated metabolic stress is accompanied by impaired neutrophil chemotaxis and NET‐associated responses in vitro, which are partially ameliorated by AZD3965. (A) Representative transmission electron microscopy images showing extracellular chromatin structures and NET‐like morphology in control, lactate‐treated (La), and lactate + AZD3965‐treated neutrophils. (B) Chemotaxis index of neutrophils in the control, La, and La + AZD3965 groups. (C) Intracellular lactate content in neutrophils from the control, La, and La + AZD3965 groups. (D) Representative immunofluorescence staining of MPO in control, La, and La + AZD3965 groups. DAPI was used for nuclear counterstaining. (E) Representative immunofluorescence staining of citrullinated histone H3 (CitH3) in control, La, and La + AZD3965 groups. DAPI was used for nuclear counterstaining. (F) Representative western blot images and densitometric analysis of MPO and CitH3 protein expression in control, La, and La + AZD3965 groups. β‐actin was used as the loading control. For quantitative analyses in (B) and (C), *n* = 6 independent biological replicates per group. For quantitative analyses in (F), *n* = 3 independent biological replicates per group. Data are presented as mean ± SEM. Multiple‐group comparisons were performed using one‐way ANOVA followed by Tukey's post hoc test. *p < 0.05 versus control; ^#^ *p* < 0.05 versus La. Scale bars as indicated.

To further evaluate the impact of lactate‐associated metabolic stress on neutrophil effector function, NET‐associated responses and chemotaxis were examined in vitro (Figure 5). TEM showed abundant extracellular chromatin structures in control neutrophils, whereas lactate‐treated neutrophils displayed markedly reduced NET‐like morphology; this effect was partially ameliorated by AZD3965 (Figure 5A). Transwell assays showed that lactate significantly reduced neutrophil chemotaxis, while AZD3965 partially ameliorated migratory capacity (Figure 5B). Intracellular lactate content was significantly increased after lactate treatment and reduced by AZD3965 (Figure 5C). Consistent with these findings, immunofluorescence and Western blot analyses showed reduced MPO and CitH3 signals in lactate‐treated neutrophils, with partial recovery following AZD3965 treatment (Figure 5D–F). Together, these findings suggest that high‐lactate‐associated metabolic stress is linked to a hyporesponsive neutrophil phenotype characterized by impaired migration and suppressed NET‐associated responses.

### 3.6 AZD3965 Partially Attenuates Lung Injury and Fibrosis‐Like Remodeling Induced by AlCl_3_ Aerosol Exposure in Vivo

**FIGURE 6:**
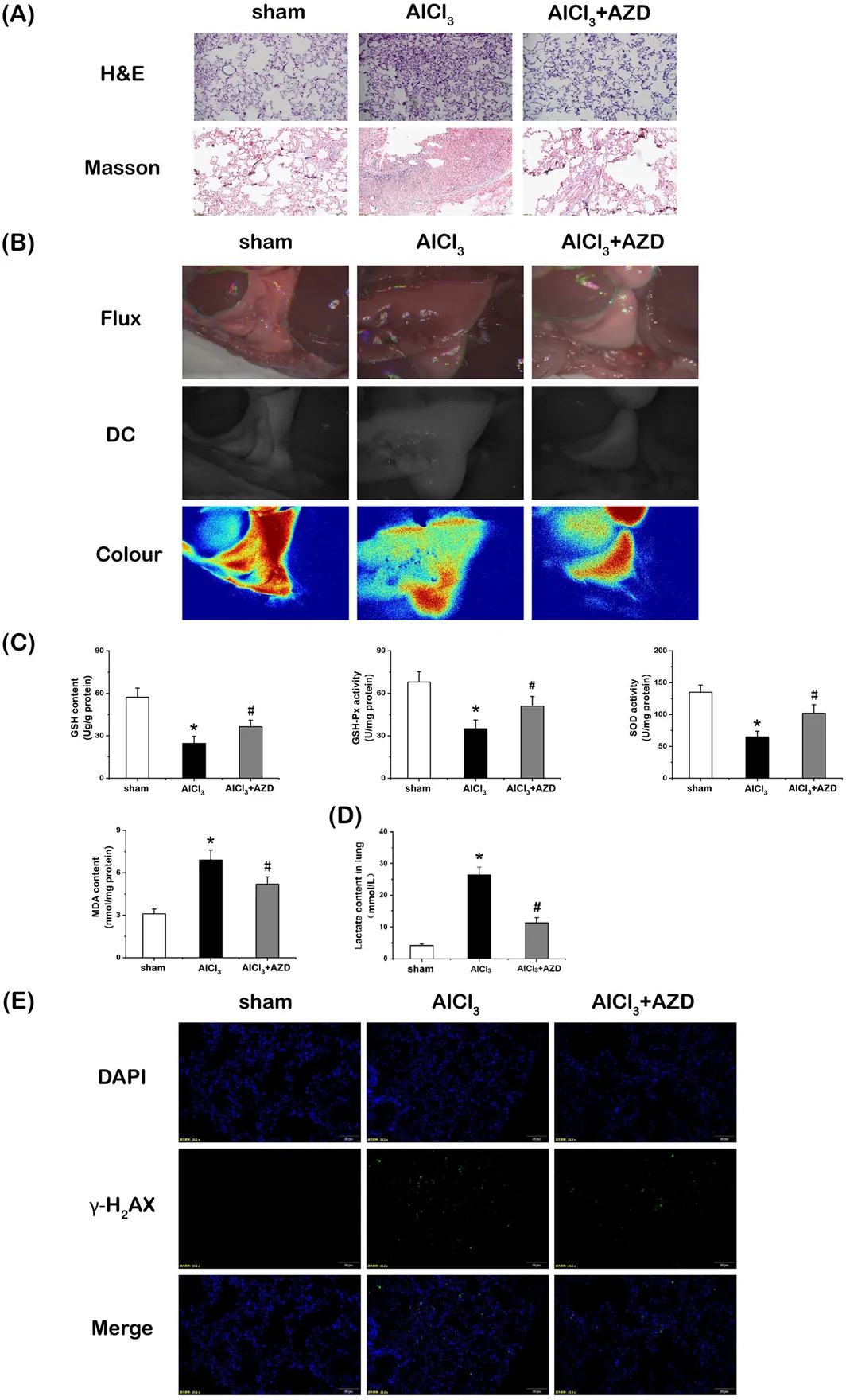
AZD3965 attenuates aluminum‐induced lung injury and fibrosis‐like remodeling in vivo. (A) Representative H&E and Masson's trichrome staining of lung sections from the sham, model, and model + AZD3965 groups. (B) Representative laser speckle contrast images showing pulmonary surface perfusion in the sham, model, and model + AZD3965 groups. (C) GSH content, GSH‐Px activity, SOD activity, and MDA levels in lung tissues from the sham, model, and model + AZD3965 groups. (D) Lung lactate levels in the sham, model, and model + AZD3965 groups. (E) Representative γ‐H2AX immunofluorescence staining showing DNA double‐strand break‐associated response signals in lung sections from the sham, model, and model + AZD3965 groups. DAPI was used for nuclear counterstaining. For quantitative analyses in (C) and (D), *n* = 6 animals/group. Data are presented as mean ± SEM. Multiple‐group comparisons were performed using one‐way ANOVA followed by Tukey's post hoc test. *p < 0.05 versus sham; #*p* < 0.05 versus model. Scale bars as indicated.

To assess the in vivo relevance of lactate‐associated pathways, rats exposed to AlCl_3_ aerosol exposure were treated with AZD3965 (Figure 6). Histological analysis showed marked alveolar structural disruption, septal thickening, and inflammatory cell infiltration in the model group, all of which were alleviated by AZD3965. Masson's trichrome staining further showed extensive collagen deposition in the model group that was reduced after AZD3965 treatment (Figure 6A). Laser speckle contrast imaging revealed impaired and heterogeneous pulmonary surface perfusion in the model group, whereas AZD3965 improved perfusion signals (Figure 6B). Aluminum aerosol exposure significantly reduced GSH content, GSH‐Px activity, and SOD activity, while increasing MDA levels; these changes were partially ameliorated by AZD3965 (Figure 6C). Lung lactate levels were markedly increased in the model group and reduced after AZD3965 treatment (Figure 6D). γ‐H2AX immunofluorescence showed increased DNA double‐strand break‐associated response signals in the model group, which were attenuated by AZD3965 (Figure 6E). These data show that AZD3965 attenuates inflammatory injury and fibrosis‐like remodeling, oxidative stress, lactate accumulation, and DNA damage response signaling induced by AlCl_3_ aerosol exposure in vivo.

### 3.7 AZD3965 Reduces Pulmonary Cytokine Production and Partially Ameliorates Neutrophil‐Associated Marker Abnormalities in Vivo

**FIGURE 7:**
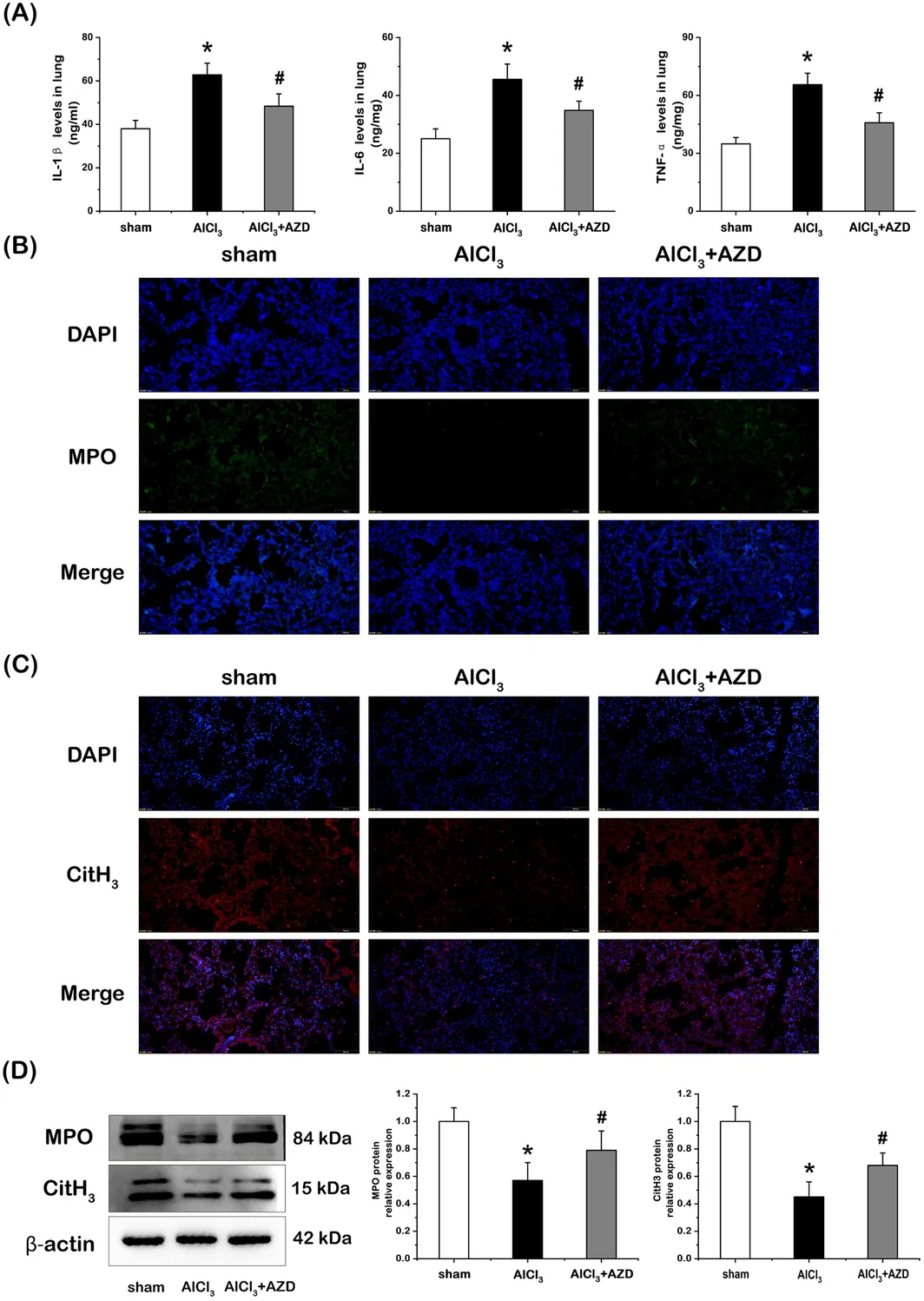
AZD3965 Reduces pulmonary proinflammatory cytokine levels and partially ameliorates neutrophil‐associated marker abnormalities in vivo. (A) Levels of IL‐1β, IL‐6, and TNF‐α in lung tissues from the sham, model, and model + AZD3965 groups. (B) Representative immunofluorescence staining of MPO in lung sections from the sham, model, and model + AZD3965 groups. DAPI was used for nuclear counterstaining. (C) Representative immunofluorescence staining of citrullinated histone H3 (CitH3) in lung sections from the sham, model, and model + AZD3965 groups. DAPI was used for nuclear counterstaining. (D) Representative western blot images and densitometric analysis of MPO and CitH3 protein expression in lung tissues from the sham, model, and model + AZD3965 groups. β‐actin was used as the loading control. For quantitative analyses in (A), *n* = 6 animals/group; in (D), *n* = 3 animals/group. Data are presented as mean ± SEM. Multiple‐group comparisons were performed using one‐way ANOVA followed by Tukey's post hoc test. *p < 0.05 versus sham; ^#^ *p* < 0.05 versus model. Scale bars as indicated.

To further examine the effects of AZD3965 on pulmonary inflammation and neutrophil‐associated responses, cytokines and NET‐related markers were assessed in lung tissue from sham, model, and model + AZD3965 groups (Figure 7). ELISA showed that IL‐1β, IL‐6, and TNF‐α levels were significantly elevated in the model group and significantly reduced after AZD3965 treatment (Figure 7A). Immunofluorescence analyses showed decreased MPO and CitH3 signals in the model group, both of which increased after AZD3965 treatment (Figure 7B,C). Western blot analysis showed a similar pattern, with reduced MPO and CitH3 protein expression in the model group and partial recovery in the model + AZD3965 group (Figure 7D). These findings suggest that AZD3965 attenuates pulmonary inflammatory activation and partially ameliorates neutrophil‐associated marker abnormalities in vivo.

### 3.8 Supportive Human Analysis Reveals Increased Parenchymal Abnormality Burden and Impaired Lung Function in Al‐ILD

**FIGURE 8:**
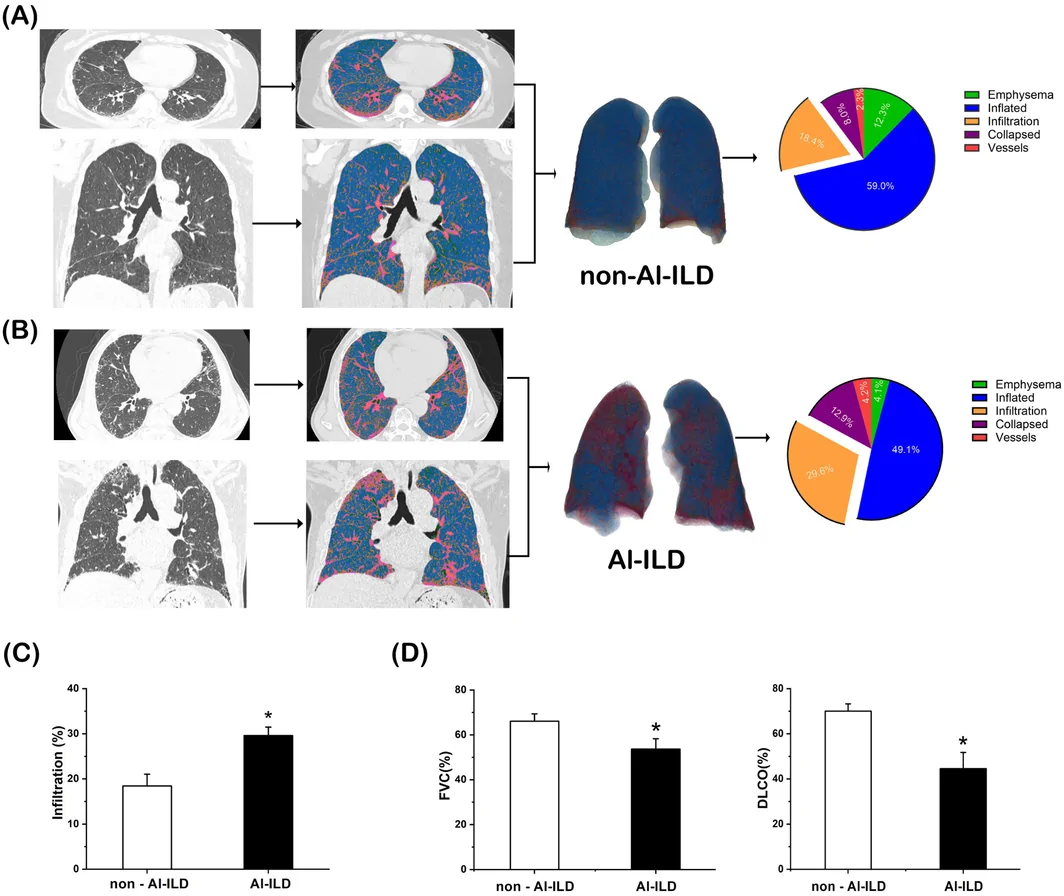
Quantitative chest CT analysis reveals increased parenchymal abnormality burden and impaired lung function in Al‐ILD patients. (A) Representative axial and coronal chest CT images from the non‐Al‐ILD group, corresponding Lung CT Analyzer‐based segmentation maps, three‐dimensional lung reconstruction, and proportional distribution of quantitative CT compartments. Segmented CT regions were classified by the software as emphysema, inflated, infiltration, collapsed, and vessels. In the present study, the software‐defined infiltration/collapsed compartments were used as an operational imaging measure of parenchymal abnormality burden. (B) Representative axial and coronal chest CT images from the Al‐ILD group, together with the corresponding segmentation maps, three‐dimensional lung reconstruction, and proportional distribution of quantitative CT compartments. (C) Quantitative comparison of the infiltration/collapsed fraction, used here as an operational imaging measure of parenchymal abnormality burden, between the non‐Al‐ILD and Al‐ILD groups. (D) Comparison of pulmonary function parameters, including forced vital capacity (FVC, % predicted) and diffusing capacity for carbon monoxide (DLCO, % predicted), between the non‐Al‐ILD and Al‐ILD groups. *n* = 8 participants in the non‐Al‐ILD group and *n* = 5 participants in the Al‐ILD group. Data are presented as mean ± SEM. Two‐group comparisons were performed using a two‐tailed Student's *t* test. *p < 0.05 versus non‐Al‐ILD.

To assess supportive human relevance, chest CT scans from patients with non‐Al‐ILD (*n* = 8) and Al‐ILD (*n* = 5) were subjected to three‐dimensional quantitative analysis using the Lung CT Analyzer module in 3D Slicer (Figure 8A,B). Representative images showed a broader distribution and greater extent of software‐defined infiltration/collapsed regions in the Al‐ILD group. Quantitative analysis demonstrated that the volumetric fraction of these compartments, used here as an operational imaging measure of parenchymal abnormality burden, was significantly increased in the Al‐ILD group compared with the non‐Al‐ILD group (Figure 8C *p* < 0.05). Pulmonary function testing further showed that FVC% predicted and DLCO% predicted were both significantly lower in the Al‐ILD group (Figure 8D *p* < 0.05). These data indicate greater structural abnormality burden and more severe functional impairment in Al‐ILD within this supportive clinical cohort.

### 3.9 Supportive Human Transcriptomics Shows Innate Immune and NET‐Related Pathway Enrichment in Al‐ILD

**FIGURE 9:**
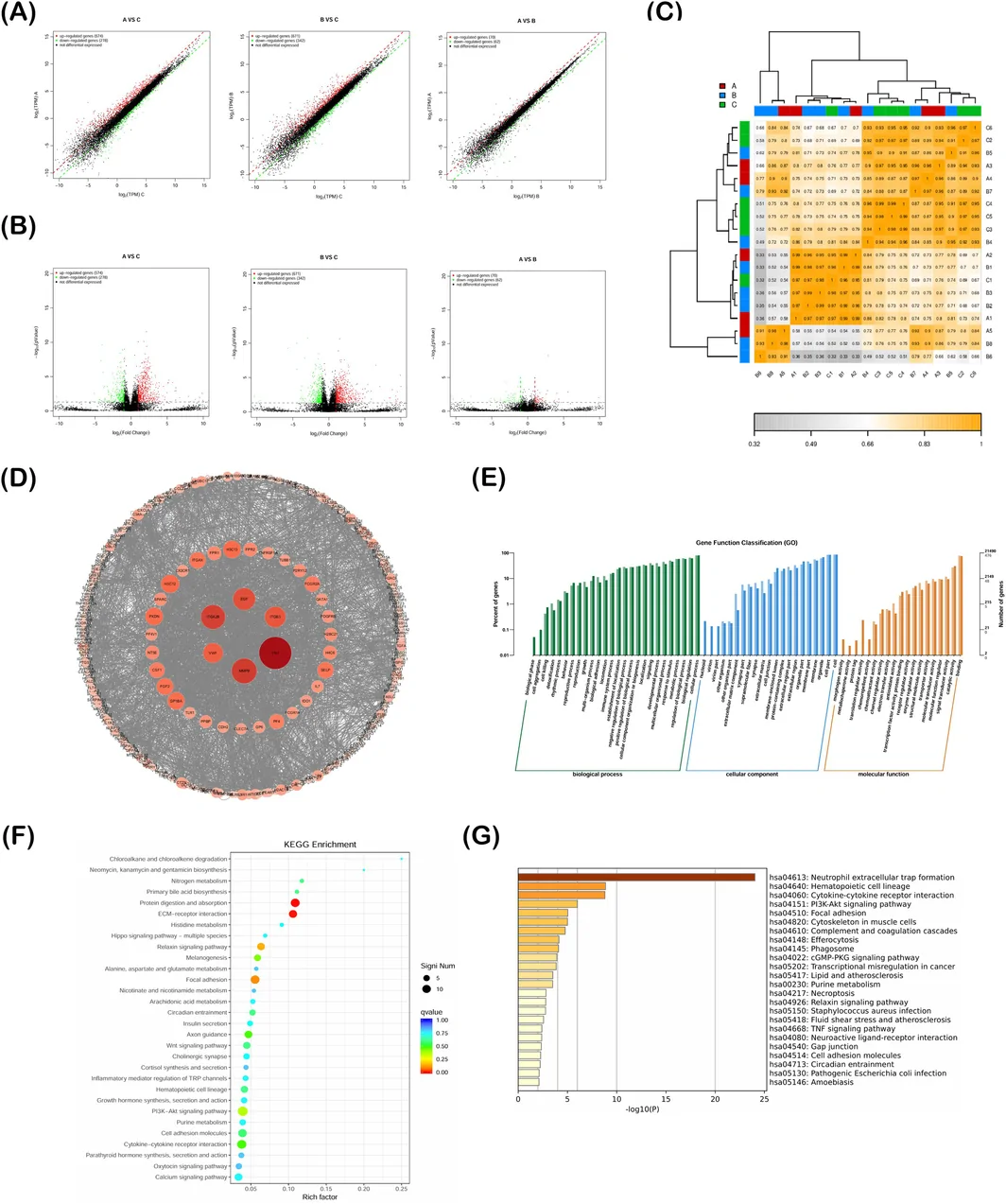
Al‐ILD exhibits a distinct transcriptomic profile enriched in innate immune, NET‐related, and inflammation‐adhesion pathways. (A) Scatter plots showing pairwise global gene expression comparisons among the Al‐ILD group (A, *n* = 5), the non‐Al‐ILD group (B, *n* = 8), and healthy controls (C, *n* = 6). (B) Volcano plots showing DEGs in pairwise comparisons among the three groups. Red indicates upregulated genes, green indicates downregulated genes, and black indicates nonsignificant genes. (C) Correlation heatmap with hierarchical clustering showing transcriptomic relationships among individual samples. (D) PPI network analysis of DEGs showing highly connected hub genes. (E) GO functional classification of DEGs across biological process, cellular component, and molecular function categories. (F) KEGG pathway enrichment analysis of DEGs. Bubble size indicates enriched gene number, and bubble color indicates adjusted significance. (G) Metascape enrichment analysis showing the top significantly enriched pathways. DEG, differentially expressed gene; GO, Gene Ontology; KEGG, Kyoto Encyclopedia of Genes and Genomes; NET, neutrophil extracellular trap; PPI, protein–protein interaction.

To further evaluate human relevance at the molecular level, blood transcriptomic profiles were compared among patients with Al‐ILD (*n* = 5), non‐Al‐ILD (*n* = 8), and healthy controls (*n* = 6) (Figure 9). Relative to healthy controls, both ILD groups showed extensive differential gene expression, and a distinct DEG pattern was also observed between the Al‐ILD and non‐Al‐ILD groups (Figure 9A,B). Pairwise correlation analysis and unsupervised hierarchical clustering showed higher within‐group similarity and segregation of Al‐ILD samples into an independent branch, supporting a distinct transcriptomic profile in Al‐ILD (Figure 9C). Protein–protein interaction analysis further showed that the DEGs formed a highly connected network containing several hub genes (Figure 9D). Functional enrichment analyses identified pathways related to extracellular matrix organization, cytokine signaling, PI3K‐Akt signaling, hematopoietic cell lineage, complement and coagulation cascades, phagosome, necroptosis, and NET formation (Figure 9E–G). These transcriptomic data provide supportive human evidence for immune, inflammatory, and remodeling programs associated with Al‐ILD.

## 4 Discussion

In the present study, we combined an AlCl_3_ aerosol exposure model, metabolomic profiling, neutrophil functional assays, and supportive human analyses to investigate the relationship between AlCl_3_ aerosol exposure, lactate accumulation, and pulmonary fibrotic remodeling. Our findings support a model in which aluminum aerosol exposure is accompanied by pulmonary lactate accumulation and neutrophil dysfunction, changes that may together contribute to persistent injury and fibrosis‐like remodeling [2, 11, 17]. This framework extends conventional oxidative stress‐centered interpretations of metal‐induced lung injury by highlighting lactate‐associated metabolic and immune dysregulation as a mechanistically relevant intermediate [17, 28, 29]. From a translational perspective, these observations also provide a rationale for considering lactate handling and neutrophil dysfunction as informative readouts in aluminum‐associated lung disease.

The animal model reproduced several features of chronic lung injury following AlCl_3_ aerosol exposure. Chronic AlCl_3_ aerosol exposure was associated with pulmonary aluminum deposition, collagen accumulation, cell death, and γ‐H2AX‐positive DNA damage response signaling, indicating persistent tissue injury accompanied by fibrosis‐like remodeling [5, 30]. Aluminum exposure also reduced antioxidant capacity and increased lipid peroxidation, consistent with sustained redox imbalance in the injured lung [31, 32]. In addition to these established injury‐related changes, metabolomic analysis identified lactate as a prominently increased metabolite in both lung tissue and serum. Given the recognized role of lactate in inflammatory and fibrotic microenvironments, this finding places lactate accumulation within the context of lung injury induced by AlCl_3_ aerosol exposure as a potentially functional component of the remodeling process, while not establishing lactate as a primary pathogenic factor [17, 28].

Our in vitro data further suggest that elevated lactate is associated with a hyporesponsive neutrophil state under high‐lactate stress conditions. Lactate reduced neutrophil viability/metabolic activity, suppressed oxidative burst, decreased mitochondrial membrane potential, weakened Ca^2+^ signaling, impaired chemotaxis, and reduced NET‐associated markers, including MPO and CitH3 [33]. Here, neutrophil dysfunction refers to a decline in defense‐, clearance‐, and repair‐related effector functions rather than enhanced inflammatory activation in the traditional sense. Rather than supporting sustained neutrophil activation, these findings indicate that excessive lactate may compromise key effector programs required for appropriate injury control and resolution. Under chronic exposure conditions, such dysfunction may favor persistent inflammation and maladaptive repair, thereby contributing to fibrosis‐like remodeling [13, 33].

Our data also provide pharmacological support for a functional contribution of lactate transport to this process. Lactate increased intracellular accumulation in neutrophils, whereas inhibition of lactate transport with AZD3965 reduced intracellular lactate levels and partially ameliorated oxidative burst, mitochondrial function, chemotaxis, and NET‐associated responses in vitro [16]. In vivo, AZD3965 attenuated inflammatory injury and collagen deposition, improved pulmonary perfusion, reduced pulmonary lactate accumulation, partially ameliorated antioxidant abnormalities, and decreased γ‐H2AX‐associated DNA damage response signals. AZD3965 also reduced IL‐1β, IL‐6, and TNF‐α levels while increasing MPO and CitH3 expression in lung tissue. Taken together, these observations are consistent with the involvement of lactate transport in lung injury following AlCl_3_ aerosol exposure; however, they should be interpreted as pharmacological support rather than definitive evidence identifying MCT1 as a disease‐specific target in this context [15]. Additional studies using genetic or cell‐specific approaches will be needed to define the precise role of MCT1 and related transporters.

NETs should therefore be interpreted as context‐dependent effector structures rather than uniformly pathogenic or uniformly protective responses. Excessive or dysregulated NET formation may amplify acute epithelial injury and inflammatory damage, whereas insufficient NET formation or impaired neutrophil effector function during chronic metabolic stress may compromise debris clearance, injury resolution, and repair coordination. Interestingly, we observed suppression rather than enhancement of NET‐associated responses under high‐lactate conditions. This differs from reports showing that lactate can promote NET release in other settings [34, 35]. Such discrepancies may reflect differences in lactate concentration, exposure duration, disease type, acute versus chronic injury stage, and neutrophil activation status. For example, short‐term lactate exposure in acutely activated neutrophils may facilitate NET formation in some infectious or inflammatory contexts, whereas sustained high‐lactate exposure may impose metabolic stress and suppress effector functions. In the present model, sustained high‐lactate exposure appears to favor neutrophil dysfunction rather than acute pro‐NET activation, a pattern that may be more relevant to chronic tissue injury and maladaptive remodeling than to short‐term inflammatory amplification [13, 36].

To explore human relevance, we analyzed a small supportive clinical cohort. Patients with Al‐ILD showed a greater CT‐defined burden of parenchymal abnormalities together with lower FVC and DLCO than patients with non‐Al‐ILD, indicating more severe structural and functional lung impairment. In parallel, blood transcriptomic profiling identified a distinct molecular program enriched for innate immune, cytokine, extracellular matrix, and NET‐related pathways. The human cohort was intentionally designed as a supportive relevance cohort. The small number of Al‐ILD cases precluded robust adjustment for exposure intensity, smoking, treatment history, ILD subtype, and disease severity. Therefore, the clinical transcriptomic and imaging findings should be interpreted as hypothesis‐supporting rather than providing independent mechanistic evidence, and pathway enrichment may partly reflect disease severity rather than an aluminum exposure‐specific mechanism.

**FIGURE 10:**
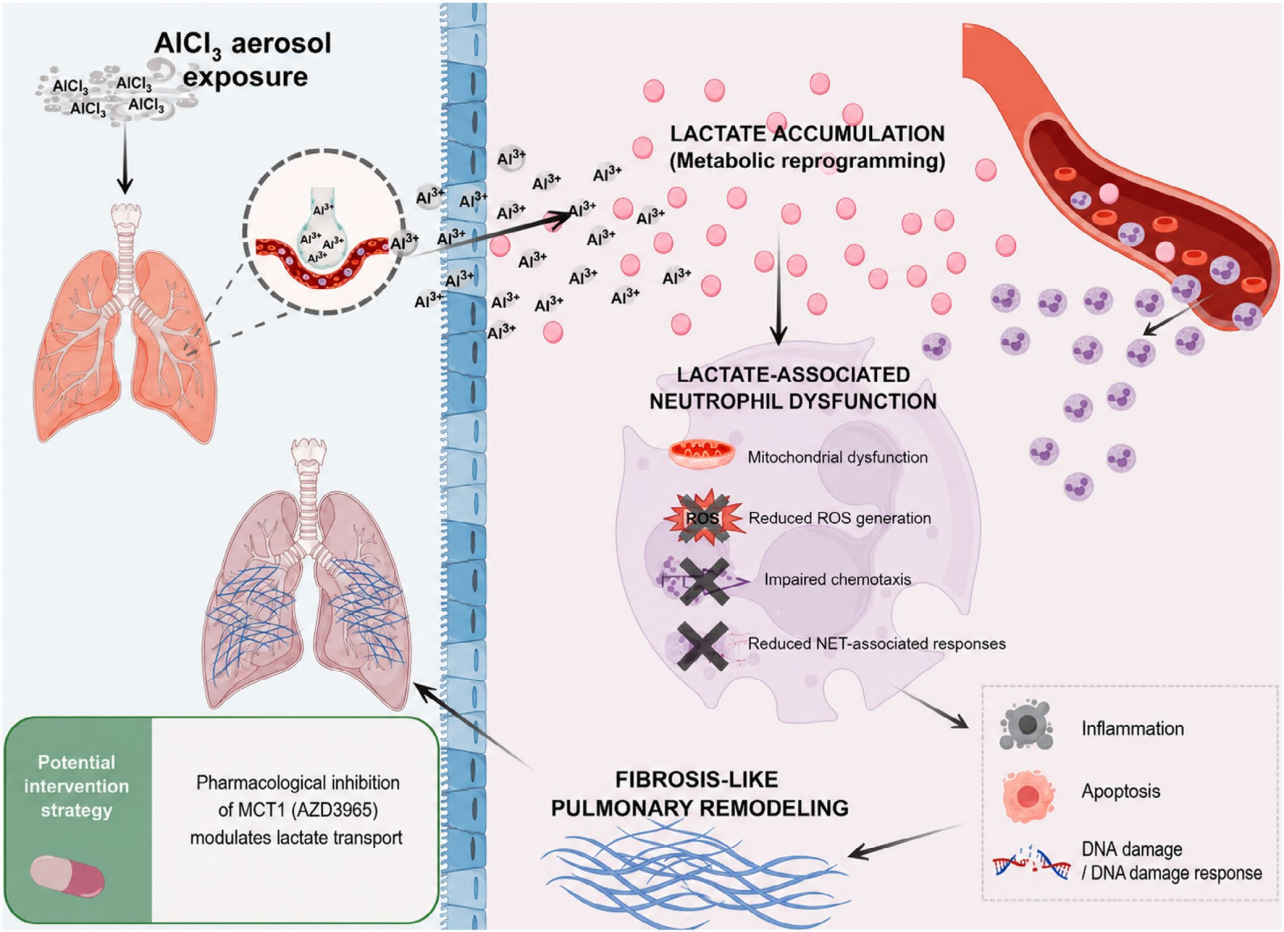
Proposed schematic framework linking environmental aluminum exposure, lactate accumulation, neutrophil dysfunction, and fibrosis‐like remodeling. Chronic inhalational exposure to aluminum‐containing particles is accompanied by pulmonary aluminum deposition and metabolic reprogramming, with lactate accumulation in the lung microenvironment. Elevated lactate is associated with neutrophil dysfunction, characterized by impaired oxidative burst, mitochondrial dysfunction, disrupted calcium homeostasis, reduced chemotaxis, and suppressed neutrophil extracellular trap (NET)–associated responses. These changes are accompanied by persistent inflammation, oxidative stress, apoptosis, and DNA damage response signaling, and may contribute to defective injury resolution and fibrosis‐like remodeling. Pharmacological inhibition of lactate transport, including MCT1 blockade by AZD3965, partially ameliorates these pathological changes, supporting the functional involvement of lactate transport in this process.

Taken together, the present findings support a mechanistic framework in which AlCl_3_ aerosol exposure is accompanied by pulmonary lactate accumulation, lactate‐associated metabolic stress may disrupt neutrophil metabolic and redox homeostasis, and neutrophil dysfunction may contribute to defective injury resolution and fibrosis‐like remodeling (Figure 10). By integrating findings from the experimental AlCl_3_ aerosol exposure model with supportive human evidence, this study highlights lactate‐associated immunometabolic dysregulation as a mechanistically relevant feature of AlCl_3_ aerosol exposure‐related lung injury and fibrosis‐like remodeling.

Several limitations should be acknowledged. First, the supportive clinical cohort was relatively small, limiting statistical power and the ability to account for exposure heterogeneity, smoking status, treatment history, disease severity, and ILD subtype differences. Second, because the human analyses were observational, they cannot independently establish causality or the temporal sequence of mechanistic events. Third, although the AlCl_3_ aerosol exposure model reproduced several relevant pathological and mechanistic features, it does not recapitulate the physicochemical complexity of occupational aluminum dust environments or heterogeneous airborne aluminum‐containing materials encountered in real‐world exposure settings [37]. An additional limitation concerns characterization of the inhalation exposure. Aerodynamic particle‐size distribution was not measured contemporaneously during the original animal exposure sessions; consequently, the experimental aerosol cannot be definitively classified as PM2.5. In addition, contemporaneous breathing‐zone concentration measurements could not be verified from the available study documentation; thus, the reported concentrations should be interpreted as target concentrations expressed as elemental aluminum equivalents. Any subsequent measurements performed under matched generation and chamber conditions should be interpreted as supportive system‐validation data rather than as contemporaneous characterization of the original exposure sessions. Future inhalation studies should include contemporaneous measurements of actual aerosol concentration at the animals' breathing zone and aerodynamic particle‐size distribution, including the mass median aerodynamic diameter, geometric standard deviation, and mass fraction at aerodynamic diameters ≤ 2.5 μm, with independent validation of real‐time monitoring. Fourth, the AZD3965‐based rescue experiments provide pharmacological support for the involvement of lactate transport but do not constitute definitive cell‐specific evidence identifying MCT1 as a disease‐specific target; the relatively high in vivo dose may also introduce nonspecific pharmacological effects. In addition, an AZD3965‐only control group and a dedicated toxicity assessment were not included; therefore, potential drug‐related effects independent of AlCl_3_ aerosol exposure cannot be fully excluded. Fifth, the 20 mM sodium L‐lactate model should be interpreted as a high‐lactate or supraphysiological stress model, and potential confounding effects beyond pH control, including osmotic pressure, sodium load, metabolic stress, and altered cellular energy status, cannot be excluded. Finally, the present study focused primarily on neutrophils and lactate transport, and additional cell populations as well as upstream sources of lactate remain to be clarified. Lactate may derive from epithelial cells, fibroblasts, immune cells, or metabolic reprogramming within injured tissue. Time‐resolved studies, lower lactate concentration gradients, isotonic or sodium salt controls, metabolic flux tracing, and single‐cell or cell‐specific approaches may help define stage‐specific immune responses, lactate sources, and cell–cell interactions during aluminum‐associated fibrotic progression.

## 5 Conclusions

In conclusion, AlCl_3_ aerosol exposure is accompanied by pulmonary lactate accumulation, neutrophil dysfunction, and fibrosis‐like remodeling. Partial amelioration by AZD3965 supports the functional involvement of lactate transport in this process. Together with the supportive radiologic, functional, and transcriptomic findings in humans, these data suggest that the lactate‐associated neutrophil dysfunction axis is a mechanistically relevant feature of the experimental AlCl_3_ aerosol exposure model and may also be relevant to aluminum‐associated human fibrotic lung disease.

## Author Contributions

**Jiayu Qin:** conceptualization, methodology, formal analysis, investigation, writing – original draft, writing – review and editing. **Fushi Dong:** methodology, formal analysis, investigation, writing – original draft. **Zhengjie Ye:** methodology, formal analysis, investigation, writing – original draft. **Xiangxin Yao:** methodology, formal analysis, investigation. **Xintong Wang:** methodology, formal analysis, investigation. **Tiquan Xiao:** methodology, software. **Jing Yang:** writing – review and editing, visualization. **Yan Zheng:** funding acquisition, software. **Yonggang Cao:** conceptualization, resources, data curation, project administration, supervision. **Chunli Che:** conceptualization, funding acquisition, resources, data curation, project administration, supervision.

## Funding

This work was supported by the Department of Science and Technology of Heilongjiang Province through the Heilongjiang Provincial “Revealing the List and Taking Command” Science and Technology Tackling Project (2022ZXJ03C01), the Natural Science Foundation of Heilongjiang Province (LH2023H019), and the Science and Technology Innovation Talent Project of the Fourth Affiliated Hospital of Harbin Medical University (HYDSYKJCXRC202120).

## Ethics Statement

This clinical study was approved by the Ethics Committee of the Fourth Affiliated Hospital of Harbin Medical University (approval no. MR‐23‐25‐002076) and was conducted in accordance with the Declaration of Helsinki and its later amendments. Written informed consent was obtained from all participants involved in the clinical study. All animal experiments were approved by the Institutional Animal Care and Use Committee (approval no. HMUDQ 20251221003) and were conducted in accordance with institutional guidelines for the care and use of laboratory animals.

## Consent

Consent for publication of de‐identified clinical data was obtained from participants where applicable.

## Conflicts of Interest

The authors declare no conflicts of interest.

## Data Availability

The datasets generated and/or analysed during the current study are available from the corresponding author on reasonable request.
